# Platelet Count in Patients with Mild Disease at Admission is Associated with Progression to Severe Hantavirus Cardiopulmonary Syndrome

**DOI:** 10.3390/v11080693

**Published:** 2019-07-30

**Authors:** René López, Cecilia Vial, Jerónimo Graf, Mario Calvo, Marcela Ferrés, Gregory Mertz, Analía Cuiza, Begonia Agüero, Dante Aguilera, Diego Araya, Ignacia Pailamilla, Flavia Paratori, Víctor Torres-Torres, Pablo A Vial, Hantavirus Study Group in Chile

**Affiliations:** 1 Departamento de Paciente Crítico, Clínica Alemana de Santiago, Santiago 7650567, Chile; 2 Escuela de Medicina. Facultad de Medicina Clínica Alemana Universidad del Desarrollo, Santiago 7710162, Chile; 3 Programa Hantavirus, Instituto de Ciencias e Innovación en Medicina (ICIM), Facultad de Medicina, Clínica Alemana Universidad del Desarrollo, Santiago 7590943, Chile; 4 Instituto de Medicina, Universidad Austral de Chile, Valdivia 5110566, Chile; 5 Departamento Enfermedades Infecciosas e Inmunología Pediátrica, Laboratorio Infectología y Virología Molecular, Facultad de Medicina, Pontificia Universidad Católica de Chile, Santiago 8331500, Chile; 6 University of New Mexico Health Sciences Center, Albuquerque, NM 87131, USA; 7 Departamento de Pediatría, Clínica Alemana de Santiago, Santiago 7650567, Chile

**Keywords:** Hantavirus cardiopulmonary syndrome, Hantavirus pulmonary syndrome, thrombocytopenia

## Abstract

Background: Hantavirus cardiopulmonary syndrome (HCPS) has a mortality up to 35–40% and its treatment is mainly supportive. A variable to predict progression from mild to severe disease is unavailable. This study was performed in patients with documented infection by Andes orthohantavirus, and the aim was to find a simple variable to predict progression to moderate/severe HCPS in patients with mild disease at admission. Methods: We performed a retrospective analysis of 175 patients between 2001 and 2018. Patients were categorized into mild, moderate, and severe disease according to organ failure and advanced support need at hospital admission (e.g., mechanical ventilation, vasopressors). Progression to moderate/severe disease was defined accordingly. Clinical and laboratory variables associated with progression were explored. Results: Forty patients with mild disease were identified; 14 of them progressed to moderate/severe disease. Only platelet count was different between those who progressed versus those that did not (37 (34–58) vs. 83 (64–177) K/mm^3^, *p* < 0.001). A ROC curve analysis showed an AUC = 0.889 (0.78–1.0) *p* < 0.001, with a platelet count greater than 115K /mm^3^ ruling out progression to moderate/severe disease. Conclusions: In patients with mild disease at presentation, platelet count could help to define priority of evacuation to tertiary care centers.

## 1. Introduction

Orthohantaviruses are enveloped, segmented, negative-sense, single stranded-RNA viruses that belong to the family *Hantaviridae* [1,2]. There are two main categories of orthohantavirus diseases related to organs involved and geographic regions: hemorrhagic fever with renal syndrome (HFRS), mainly in Asia and Europe, caused by “Old World” orthohantaviruses, and hantavirus cardiopulmonary syndrome (HCPS), in North and South America, caused by “New World” orthohantaviruses [3].

Andes orthohantavirus (ANDV) is endemic in Chile and Argentina, and its main reservoir is the long-tailed pygmy rice rat (*Oligoryzomys longicaudatus*) [4]. Transmission to humans occurs primarily by inhalation of the virus in aerosolized rodent excretions, and people living in rural areas or routinely performing activities in these locations such as farmers, forest workers or people that engage in recreational activities in endemic places have a higher risk of becoming infected [5,6]. Person-to-person transmission has been documented only for ANDV infection [7,8,9,10]. Moreover, sexual partners and contacts who slept in the same bed during the prodromal period of the case had ten times more risk of becoming infected when compared to other household contacts [9,10].

The incubation period of New World orthohantaviruses varies from 7 to 49 days [11,12], and viral RNA can be detected in blood as early as two weeks prior to symptoms or antibody response [9]. Patients may progress to a severe disease that starts after 3 to 6 days of a prodromal phase characterized by nonspecific symptoms such as fever, malaise, headache, and myalgia and sometimes with gastrointestinal disorders. The prodrome is followed by respiratory symptoms that evolve from dry cough to respiratory failure due to capillary leak into the pulmonary interstitium, evidenced by chest radiographs showing peribronchial haze and Kerley’s B lines that subsequently progresses to alveolar flooding [3,13]. HCPS also may present with circulatory shock, with cardiovascular depression in a high proportion of cases [1,14,15].

With fatality rates of 35–40%, HCPS is one of the deadliest infectious diseases; unfortunately, there are no drugs with proven efficacy for HCPS. Treatment is based on critical care support, including extracorporeal membrane oxygenation (ECMO) [16]. One open treatment study published in 2015 showed that the administration of human immune plasma with anti-ANDV neutralizing antibodies appeared to be safe when administered in confirmed or suspected HCPS with a reduction in case fatality rate [17].

Early diagnosis during the febrile prodrome is quite difficult due to the lack of specific initial symptoms or specific diagnostic findings with routine laboratory tests [3,13,18]. Only after onset of the cardiopulmonary phase are characteristic hematological parameters present, including hemoconcentration, thrombocytopenia, elevated white blood cell count, immature granulocytes and distinctive immunoblast cells [19]. The presence of immunoblasts (> 10%) has shown 25% sensitivity with 98% specificity; while thrombocytopenia has exhibited 98% sensitivity with 74% specificity [20]. Moreover, anti-orthohantavirus antibodies are usually negative until late in the febrile prodromal or the beginning of cardiopulmonary phase [9,18]. In recent years, the use of two-step quantitative reverse-transcription polymerase chain reaction (PCR) in peripheral blood nucleated cells as a diagnostic tool for ANDV has been validated allowing early and fast viral detection [18].

At present, in patients with initially mild orthohantavirus disease in the cardiopulmonary phase, a variable to predict progression to severe disease (respiratory failure and/or shock with advanced support requirement) is not available. In Chile, the protocol of public health authority includes the early evacuation of all patients with orthohantavirus infection (independent of their clinical severity) to tertiary centers with ICU and ECMO availability [19]. To optimize health resource and evacuation priority, a mechanism to predict progression to severe disease would be helpful.

The aim of this study was to find a simple variable to predict progression to moderate or severe HCPS for a group of initially mild Andes orthohantavirus cases.

## 2. Materials and Methods

### 2.1. Study Design and Patients

This is an observational, retrospective and analytical study. The cohort was composed of patients from a prospectively obtained database by the Hantavirus Program from the Instituto de Ciencias e Innovación en Medicina de la Facultad de Medicina, Clínica Alemana-Universidad del Desarrollo. All primary data considered for this study were collected prospectively from 2001 until 2018, in 5 cohorts of patients enrolled for subsequent studies in 12 health centers from 9 Chilean cities.

The diagnosis of HCPS was confirmed in all cases by quantitative enzyme-linked immunosorbent assay (ELISA) detecting ANDV specific immunoglobulin M. In some cases (*n* = 100) diagnosis was also made by reverse-transcription PCR detecting ANDV RNA. Even though ELISA and PCR cross-reactivity between orthohantaviruses is well described, substantial numbers of viruses have been sequenced in Chile and all have been ANDV [4]. It is reasonable then to assume that the cases resulted from ANDV infection.

Demographic, clinical, laboratory and hospital mortality data were collected using a standardized case record form, and deidentified data was entered into a dedicated database. According to clinical presentation, patients were categorized as mild, moderate or severe diseases, defined as follow:

Mild disease: Patients with prodromal symptoms without respiratory failure (defined as PaO_2_ <60 mmHg at FiO_2_ 21%).

Moderate disease: Patients with organ dysfunction but without requirement of advanced support (defined as the requirement of inotropic and/or ventilatory support).

Severe disease: Patients that required advanced life support.

The group of patients with ANDV infection that progressed from mild disease to moderate or severe disease was defined as “progression” or “progressive disease”. Patients who remained as mild disease along their hospital stay were defined as “non-progression” or “non-progressive disease”.

The institutional ethical board approved this non-interventional study with anonymized data and waived the informed consent requirement.

### 2.2. Patients of Interest

We performed a demographic and laboratory characterization of all patients. Patients in the cardiopulmonary phase with mild disease at admission were selected and categorized in two groups as follow: a) Patients who progressed to moderate/severe disease and b) patients who did not progress to moderate/severe disease. We defined the prodromal time as the number of days with fever before hospital admission. Prodromal time, demographic, and laboratory variables were compared according to clinical progression of disease during the hospital stay. If continuous variables were different between patients with and without disease progression, a cutoff was identified.

### 2.3. Outcomes

The primary outcome was the progression to moderate/severe disease. Secondary outcome was in-hospital mortality.

### 2.4. Statistics

An initial descriptive analysis was performed. Categorical variables are shown as number of patients with percentage in parentheses and compared with Fisher’s exact test. Continuous variables are expressed as median (IQR) and compared with Mann Whitney U test. A cutoff value was determined for continuous variables associated with progression of disease using receiver operating characteristic (ROC) curve analysis. A Bayesian analysis was then performed using this cutoff value. Odds ratios (OR) with confidence intervals were obtained when possible. Significance was defined as *p* value < 0.05. Statistical analysis was performed using the SPSS software, version 20 (SPSS, Chicago, IL, USA).

## 3. Results

A total of 175 patients with ANDV infection were identified. They were mainly young males with a median hospital length of stay (LOS) of 10 days and hospital mortality rate of 21%. The demographic, clinical and laboratory data are shown in Table 1.

Almost a forth of patients (*N* = 40) had mild orthohantavirus disease at hospital admission. Fourteen of them had progression in clinical severity during hospital stay, including four patients who died. Specifically, two patients progressed to moderate disease while twelve progressed to severe disease. Detailed data of these patients is shown in Table 2. In patients with progression, a significantly lower platelet count was observed at admission.

Attending to the significant association between platelet count and disease progression, we explored possible associations between platelet count and other laboratory variables at admission using the Pearson’s correlation coefficient. Platelet count was inversely correlated only to the hematocrit level (Table 3).

**Figure 1:**
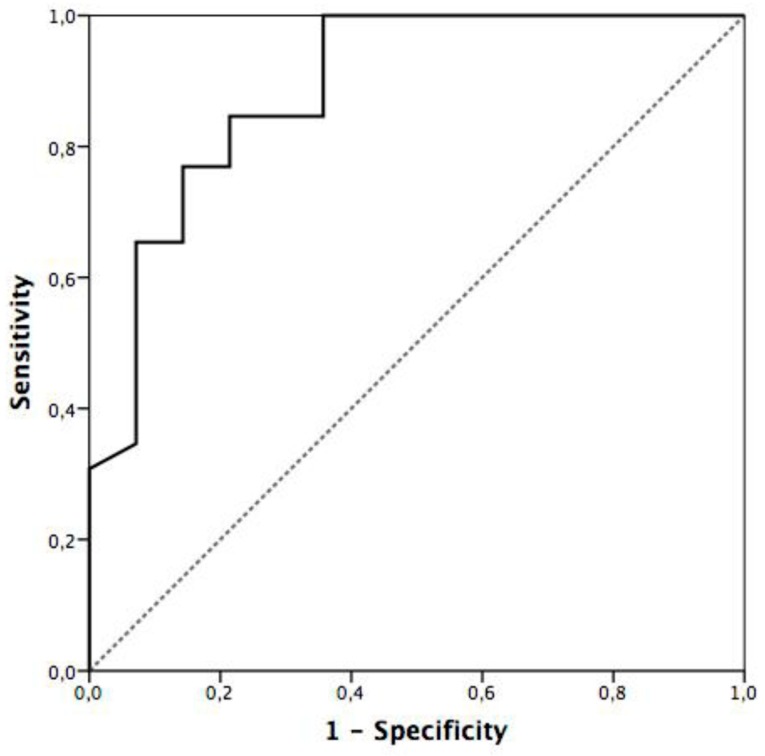
Receiver operating characteristic (ROC) curve to assess the ability of platelet count at admission to identify patients with progressive and non-progressive disease. Area under the curve (AUC) was 0.889 (0.78–1.0), *p* < 0.001.

When the platelet count’s ability to identify patients with progressive and non-progressive disease is explored using a ROC curve (Figure 1), a good performance is observed with an AUC = 0.889 (0.78–1.0) *p* < 0.001.

A Bayesian analysis was performed with data from ROC curve analysis of platelets. We observed that a platelet count higher than 115K at admission ruled-out progression to moderate/severe disease, with a sensitivity of 0.31 and specificity of 1 (Table 4).

On the other hand, patients with a platelet count lower than 40K have a significantly higher probability to progress to moderate/severe disease, with an OR = 70 (4–1362), *p* = 0.005.

## 4. Discussion

The main findings of this study are that within a large cohort of ANDV HCPS cases with an overall mortality of 21%, almost a quarter of the patients presented with mild disease at admission. Among these patients with mild disease at admission, one third progressed to moderate/severe disease, most of them required ICU support, and 10% of them died. Among patients with mild disease at admission, only the platelet count was significantly different between progressive and non-progressive disease patterns; a platelet count higher than 115 K/mm^3^ at admission was a good indicator of non-progressive disease, while a platelet count lower than 40 K/mm^3^ carried a significant risk of death. The only parameter significantly associated with the platelet count at admission was the hematocrit which was inversely correlated, but did not provide prognostic information for those presenting with mild disease.

The findings in peripheral blood analysis observed in this cohort are in agreement with previously reported data [20,21]; therefore, hemoconcentration and thrombocytopenia remain important laboratory findings for HCPS diagnosis consideration [3,13,20,21]. Overall mortality of this cohort is in agreement with previous reports of HCPS, though in the lower end of the spectrum [15,16,17,22].

In septic critically ill patients, thrombocytopenia has been associated with poor outcomes [23]. In our study, in contrast, thrombocytopenia was observed early, even before the onset of the critical phase. A prognostic role for thrombocytopenia in the HFRS has been suggested. In patients with HFRS caused by Hantaan orthohantavirus infection, the platelet distribution width at admission was reported as a mortality risk factor [24], while an independent association between nadir platelet count and development of severe acute kidney injury has also been described in these patients [25]. Interestingly, thrombocytopenia has been associated with capillary leakage and severity of inflammation in Puumala orthohantavirus infection [26]. In these patients, increased thrombopoiesis and platelet activation with intravascular coagulation have also been reported [27].

The mechanisms by which orthohantaviruses cause capillary leak and thrombocytopenia are only partially understood. Both ANDV and Hantaan virus have the ability to recruit quiescent platelets to the infected endothelial cell surface through a β3 integrin-dependent mechanism [28]. A platelet layer then covers the surface of infected endothelial cells and has the potential to alter endothelial cell functions that affect vascular permeability [28]. Within the pulmonary microvasculature platelet covered endothelial cells might impair gas exchange, contribute to hypoxia and hypoxia-inducible factor 1α-mediated vascular endothelial growth factor induction which causes permeability pulmonary edema. Thrombocytopenia, hypoxia and capillary leak may, therefore, be linked through dysregulation of endothelial β3 integrin functions in othohantavirus infections [29].

Thrombocytopenia is a laboratory trait of HCPS; however, this is the first study in which an association is found between early thrombocytopenia and outcomes. Furthermore, with a “safe” platelet count cutoff of 115 K/mm^3^, progression to moderate/severe disease was excluded. If these results were confirmed, the application of such a cutoff could reduce the evacuation of patients with mild orthohantavirus diseases to centers with ECMO availability by 20%. On the other end, when the platelet count was lower than 40 K/mm^3^ an increased risk of disease progression was observed. This “severity” cutoff could help to prioritize patients in who evacuation may be mandatory. Our findings are in contradiction with other reports that did not show any relationship between platelet count and HCPS severity or mortality [14,30]. This apparent contradiction could be explained by our novel severity stratification at admission and the use of ROC curve analysis to establish a specific platelet threshold value independent of the “normal” laboratory range (usually 150 K–400 K/mm^3^).

Some limitations of this study are its retrospective nature and a relatively low number of mild orthohantavirus disease patients. A pertinent consideration is the comparatively low incidence of HCPS with respect to other infectious diseases. Also, other variables of interest may be considered in a future study (heart rate, respiratory rate, body temperature, troponins, natriuretic peptides, etc.). However, this hypothesis generator study establishes the basis for a future prospective exploration with special attention on platelets. Among the strengths of our study are the relatively high total number of patients with confirmed HCPS analyzed, the detailed characterization with availability of admission condition and clinical progression along the hospital stay, and the focus on simple and broadly available variable to assess risk stratification in these patients.

We prudently recommend that these findings should not be directly applied in clinical decision making until a validation study, in a different cohort, is performed.

## 5. Conclusions

Among patients with mild ANDV disease at admission, 35% progressed to moderate/severe disease and 10% died. Progression from mild to moderate/severe disease was not observed when the platelet count was higher than 115 K/mm^3^ at admission. In contrast, a significant increase in the risk of clinical progression was observed when the platelet count was lower than 40 K/mm^3^ at admission.

## Figures and Tables

**Table 1:** Characterization of all patients considered in this study (*N* = 175). SP (systolic arterial pressure), DP (diastolic arterial pressure), P/F ratio (arterial oxygen partial pressure to inspired oxygen fraction ratio), LDH (lactate dehydrogenase), ALT (alanine aminotransferase), AST (aspartate aminotransferase). Prodromal time was defined as the number of days with fever before hospital admission. The proportion of patients with mild, moderate and severe disease at admission is presented. Severity during hospital stay is the maximal disease category achieved along the in-hospital course according to the same three categories defined in methods. Hospital length of stay (LOS) and in-hospital mortality are also included.

Variables	Value	Reference Value
Age, years, median (IQR)	35 (23–46)	
Male, N (%)	123 (70)	
Prodromal time, days, median (IQR)	5 (4–7)	
SP, mmHg, median (IQR)	108 (99–120)	
DP, mmHg, median (IQR)	66 (60–74)	
P/F ratio, median (IQR)	167 (114–248)	
Lactate, mmol/L, median (IQR)	1.8 (0.3–3.3)	0.8–1.7
Hematocrit, %, median (IQR)	44 (39–49)	37–47
Leukocytes, x1000/mL, median (IQR)	11.5(8.1–17.4)	4–10
Platelets, x1000/mL, median (IQR)	56 (37–86)	150–400
LDH, U/L, median (IQR)	756 (477–1100)	105–333
Blood pH, median (IQR)	7.42 (7.34–7.45)	7.35–7.45
Serun creatinine, mg/dL, median (IQR)	0.9 (0.7–1.4)	0.6–1.2
ALT, U/L, median (IQR)	66 (44–117)	5–37
AST, U/L, median (IQR)	110 (71–197)	10–41
Amylase, U/L, median (IQR)	44 (34–71)	0–125
Respiratory failure, N (%)	133 (77)	
Invasive mechanical ventilation, N (%)	87 (51)	
Circulatory failure, N (%)	71 (41)	
**Severity at Admission**		
Mild disease, N (%)	40 (23)	
Moderate disease, N (%)	86 (49)	
Severe Disease, N (%)	49 (28)	
**Severity during Hospital Stay**		
Mild disease, N (%)	26 (14)	
Moderate disease, N (%)	56 (32)	
Severe Disease, N (%)	94 (54)	
**Hospital LOS, Days, Median (IQR)**	10 (7–16)	
**In-hospital Mortality, N (%)**	36 (21)	

**Table 2:** Comparison of patients with progressive disease (*N* = 14) versus non progressive disease (patients who remain as mild disease during hospital stay, *N* = 26). SP (systolic arterial pressure), DP (diastolic arterial pressure), P/F ratio (arterial oxygen partial pressure to inspired oxygen fraction ratio), LDH (lactate dehydrogenase), ALT (alanine aminotransferase), AST (aspartate aminotransferase), Hospital LOS (hospital length of stay). Prodromal time was defined as the number of days with fever before hospital admission. Only platelet count was different between both groups of patients (Mann Whitney U Test). Mortality was higher in patients with progressive disease (Fisher’s exact test).

Variables	All *N* = 40	Progression *N* = 14	Non-Progression *N* = 26	Significance
Age, years, median (IQR)	38 (26–46)	36 (27–46)	39 (28–46)	0.827
Male, N (%)	32 (80)	12 (86)	20 (77)	0.412
Prodomal time, days, median (IQR)	4 (3–5)	3 (3–6)	4 (4–5)	0.278
SP, mmHg, median (IQR)	113 (100–128)	105 (100–122)	117 (100–129)	0.406
DP, mmHg, median (IQR)	67 (60–71)	65 (60–76)	70 (60–70)	0.944
Lactate, mmol/L, median (IQR)	1.8 (1.1–2.3)	1.9 (1.6–3.0)	1.4 (1.1–2.2)	0.299
Hematocrit, %, median (IQR)	44 (44–48)	44 (40–47)	43 (38–48)	0.285
Leukocytes, x1000/mL, median (IQR)	9.8 (6.7–12.4)	10.9 (9.4–16.1)	8.7 (6.3–11.8)	0.104
Platelets, x1000/mL, median (IQR)	71 (44–110)	37 (34–58)	83 (64–127)	<0.001
LDH, U/L, median (IQR)	779 (485–1008)	726 (486–1593)	798 (483–978)	0.432
Blood pH, median (IQR)	7.43 (7.39–7.47)	7.40 (7.33–7.45)	7.45 (7.41–7.48)	0.149
Serum creatinine, mg/dL, median (IQR)	0.9 (0.5–1.2)	1.0 (0.4–1.3)	0.9 (0.4–1.0)	0.860
ALT, U/L, median (IQR)	51 (33–90)	46 (30–82)	53 (33–129)	0.225
AST, U/L, median (IQR)	99 (54–177)	135 (52–175)	87 (56–186)	0.731
Amylase, U/L, median (IQR)	42 (33–66)	38 (31–57)	44 (34–69)	0.521
Hospital LOS, days, median (IQR)	9 (6–14)	12 (7–18)	9 (6–12)	0.154
In-hospital mortality, N (%)	4 (10)	4 (29)	0 (0)	0.011

**Table 3:** Correlations between platelet count and other laboratory variables at hospital admission. We only found a significant correlation between platelet count and hematocrit level. LDH (lactate dehydrogenase).

Variables	R	P Value
Prodomal time	−0.172	0.481
Lactate	−0.461	0.153
Hematocrit	−0.394	0.012
Leukocyte count	−0.064	0.697
LDH	−0.345	0.084
Blood pH	−0.006	0.974
Serum creatinine	0.14	0.417

**Table 4:** 2 × 2 contingency table for progression to moderate/severe Hantavirus cardiopulmonary syndrome (HCPS) of Andes orthohantavirus (ANDV) mild disease cases at admission versus a platelet count threshold of 115 K/mm^3^ at admission.

		Disease during Hospital Stay		
		Progression	Non-Progression	Total
Platelets	<115K	14	18	32
	>115K	0	8	8
		14	26	40
